# Independent development of the Reach and the Grasp in spontaneous self-touching by human infants in the first 6 months

**DOI:** 10.3389/fpsyg.2014.01526

**Published:** 2015-01-08

**Authors:** Brittany L. Thomas, Jenni M. Karl, Ian Q. Whishaw

**Affiliations:** Department of Neuroscience, Canadian Centre for Behavioural Neuroscience, University of LethbridgeLethbridge, AB, Canada

**Keywords:** reach, grasp, prehension, self-touch, sensorimotor development, development of reaching, development of grasping

## Abstract

The Dual Visuomotor Channel Theory proposes that visually guided reaching is a composite of two movements, a Reach that advances the hand to contact the target and a Grasp that shapes the digits for target purchase. The theory is supported by biometric analyses of adult reaching, evolutionary contrasts, and differential developmental patterns for the Reach and the Grasp in visually guided reaching in human infants. The present ethological study asked whether there is evidence for a dissociated development for the Reach and the Grasp in nonvisual hand use in very early infancy. The study documents a rich array of spontaneous self-touching behavior in infants during the first 6 months of life and subjected the Reach movements to an analysis in relation to body target, contact type, and Grasp. Video recordings were made of resting alert infants biweekly from birth to 6 months. In younger infants, self-touching targets included the head and trunk. As infants aged, targets became more caudal and included the hips, then legs, and eventually the feet. In younger infants hand contact was mainly made with the dorsum of the hand, but as infants aged, contacts included palmar contacts and eventually grasp and manipulation contacts with the body and clothes. The relative incidence of caudal contacts and palmar contacts increased concurrently and were significantly correlated throughout the period of study. Developmental increases in self-grasping contacts occurred a few weeks after the increase in caudal and palmar contacts. The behavioral and temporal pattern of these spontaneous self-touching movements suggest that the Reach, in which the hand extends to make a palmar self-contact, and the Grasp, in which the digits close and make manipulatory movements, have partially independent developmental profiles. The results additionally suggest that self-touching behavior is an important developmental phase that allows the coordination of the Reach and the Grasp prior to and concurrent with their use under visual guidance.

## Introduction

The Dual Visuomotor Channel theory proposes that visually guided reaching consists of two movements, the Reach and the Grasp, each mediated by separate visuomotor pathways from occipital to parietofrontal neocortex (Arbib, 1981; Jeannerod, 1981, 1999; Rizzolatti et al., 1998; Tanné-Gariépy et al., 2002; Culham and Valyear, 2006; Cavina-Pratesi et al., 2010; Filimon, 2010; Karl and Whishaw, 2013). The Reach transports and orients the hand in relation to the extrinsic (location) features of a target while the Grasp opens, shapes, and closes the hand for target purchase in relation to the intrinsic (size, shape) features of the target. Visual fixation of a target from movement onset to target contact integrates the Reach and the Grasp into a seamless act (de Bruin et al., 2008; Sacrey and Whishaw, 2012). In a number of situations in which online vision is not available to guide reaching, the Reach and the Grasp can become uncoupled, each becoming directed by somatosensory guidance. Proprioception guides the Reach to locate the target whereas the Grasp is initiated from information obtained after the target is touched (Karl et al., 2012a; Karl and Whishaw, 2013; Hall et al., 2014). Visually guided reaching is likely accomplished through the same parietofrontal Reach and Grasp pathways that mediate somatosensory guided reaching (Dijkerman and de Haan, 2007; Fiehler et al., 2009; Fiehler and Rösler, 2010; Karl et al., 2012b). In short, anatomical, electrophysiological, brain imaging and behavioral evidence provide support for the idea that reaching consists of two movements, the Reach and the Grasp, which can be configured in various ways depending upon the availability of sensory guidance from different sensory systems.

At the present time, little is known about how the Reach and the Grasp become integrated as a seamless visually guided act but it is reasonable to suppose that development in infancy plays a formative role. A number of prereach and pregrasp movements displayed by infants at different stages of development can be viewed as supporting the idea that the Reach and the Grasp have independent developmental origins. Prior to the onset of visually guided reaching, prereach movements include first orienting the eyes and head to a visual target (Greenman, 1963; Kremenitzer et al., 1979; von Hofsten and Rosander, 1997), then reaching for an object with the mouth by thrusting the head forward and flexing the abdominals (Foroud and Whishaw, 2012), and eventually swiping at a visual target with a fisted or open hand (White et al., 1964; von Hofsten, 1982, 1984). Pregrasp movements include orienting the hand to, and closing the fingers on, an object that contacts the hand (Twitchell, 1965), performing spontaneous hand and grip configurations during vacuous hand babbling (Wallace and Whishaw, 2003), and manipulating objects (Lobo et al., 2014). Some prereach and pregrasp movements likely begin in utero (Myowa-Yamakoshi and Takeshita, 2006). The descriptions of these prereach and pregrasp movements indicate that they are not only made in relation to visual stimuli but they are importantly associated with somatosensory stimulation derived from hand contact with a target (Lockman et al., 1984; Newell et al., 1993; Corbetta et al., 2014).

One prediction of the Dual Visuomotor Channel theory of reaching is that development should feature independence in the maturation of the Reach and the Grasp. Indeed, a number of previous lines of investigation have noted that reaching without grasping occurs at an earlier developmental age than reaching with grasping (Von Hofsten and Lindhagen, 1979; von Hofsten, 1984; Savelsbergh and van der Kamp, 1994; Wimmers et al., 1998a,b). Nevertheless, there are divergent predictions related to the significance of the independence of behaviors described as reaching and grasping. For example, catastrophe theory proposes that during development, reaching gives way to grasping and that the transition point or cusp is associated with enabling morphological changes such as those of hand size, arm size, and torso strength (Wimmers et al., 1998a,b). In contrast, Dual Visuomotor Channel theory would favor the idea that the Reach and Grasp remain independent but that development also fosters conditions in which they can be combined, as occurs when the Reach and the Grasp are integrated together under online visual or somatosensory guidance (Karl and Whishaw, 2013; Corbetta et al., 2014).

Many of the studies that have investigated infant reaching have focused on visually guided reaching and so have used older infants that display visually guided reaching and grasping. Somatosensory guided reaching has received less study (but see Corbetta et al., 2014). The present study was prompted by the observation by Wallace and Whishaw (2003) that at approximately 4 months of age there is a decrease in the spontaneous vacuous arm and hand movements made by infants that is seemingly replaced by self-grasping of the body and clothing. These self-grasping movements have not received experimental analysis and we hypothesized that they could provide insights into the development of infant reaching behavior and the organization of visuomotor systems. First, they would indicate whether there is a phase of somatosensory-related reaching/grasping that precedes and/or is integrated with the onset of visually guided reaching. Second, the analysis of these movements could provide further support for the theory that the Reach and the Grasp are behaviorally independent but can be integrated through experience. Third, analysis of these movements could test the notion that the Reach and the Grasp are supported by at least partially independent neural channels. The present ethological study was therefore directed toward characterizing self-touching behavior in developing human infants over the first 6 months of life.

An important feature of the analysis included determining the relationship between infant age, the location of hand contact, and the type of hand-to-body contact. Accordingly, self-touching movements were coded in relation to the part of the hand that contacted the body (i.e., Dorsum—side or back of the hand, or Palmar—digit surface and palm) and the location on the body at which the contact was made (i.e., Rostral—head or torso, or Caudal—legs or feet). In addition, any self-grasping movement with a digit or number of digits on the body or clothes was also documented. Video recordings of the infants were made across the first 6 months of life because this time period includes the age at which self-grasping movements have been documented and precedes the age at which visually guided reaching becomes a frequent infant activity.

## Materials and methods

### Research participants

Forty-two normal, full term infants (21 boys and 21 girls) participated in the study. None of the infants had sensory or motor impairments. The initial observations were made within a few days of birth and filming sessions ended when the infants were approximately 24 weeks old (Wallace and Whishaw, 2003). This period precedes the age at which visually guided reaching becomes pronounced.

Infants were recruited from acquaintances of the authors, private day homes, the University of Lethbridge Daycare, and a local Montessori preschool (Sacrey et al., 2012). The daycare, preschool, and day homes provided the age of the child in weeks to the experimenters. Informed consent was obtained from the parent(s) prior to their child participating in the study. The University of Lethbridge Human Subjects Research Committee approved the study. All parents were naïve to the purpose and hypothesis of the study.

### Video recording

Participants were recorded using a Sony Hi8 video camera, a Sony MiniDV video camera, or a Casio Exilim digital camera. All Hi8 and MiniDV tapes were converted to digital formats. The scorers analyzed the video recordings using slow-motion playback on QuickTime Player 7.

### Filming procedure

For filming, the infants were either lying on their back or sitting in baby seats, with the older infants usually supported in a baby seat or sometimes supported by a parent (see Lobo and Galloway, 2013, **Figure 2** for illustration of infant supported in baby seat). The seating arrangement was in part determined by parental transport preference. Nevertheless, because Savelsbergh and van der Kamp (1994) have found that body orientation to gravity influences early infant reaching, as does the location of target objects relative to the upper and lower visual fields, every attempt was made to maintain a relatively constant body orientation for the participants across the study period.

The infants were required to be unencumbered by long cuffs that covered the hands or blankets that covered their hands, body, or legs. The infants were filmed from a front view in such a way that the entire infant was visible. This necessitated placing the camera above infants that were lying on their back and before infants that were sitting. The infants did not have toys or other objects present that would otherwise distract them from spontaneous activity.

### Data collection

At least 10 min duration of spontaneous activity was filmed for an infant on each filming session. At each sampling age, between 8 and 10 infants comprise the final data set. Some of the infants were available for repeated filming (*n* = 4 for all sessions), whereas others were filmed at only a few time points. There were no obvious differences in the data obtained from infants that were repeatedly filmed and those that were filmed only once. The samples were taken as close as possible to the 2 week interval markers (i.e., when the infant was exactly 2 weeks old, 4 weeks old, etc.) as long as the infants were alert during these recordings.

### Scoring

The actions of both hands were coded separately. Because no differences in the frequencies of the types of movements were found between the two hands, the results from the two hands were combined for analyses. The infants made a large number of arm and hand movements during the recording sessions, but only punctuate contacts by the hand with the body were subject to analysis. Hand contacts were classified according to contact location (Rostral or Caudal body contacts) and hand posture (Dorsum, Palmar, or Grasp contacts).

**Figure 1:**
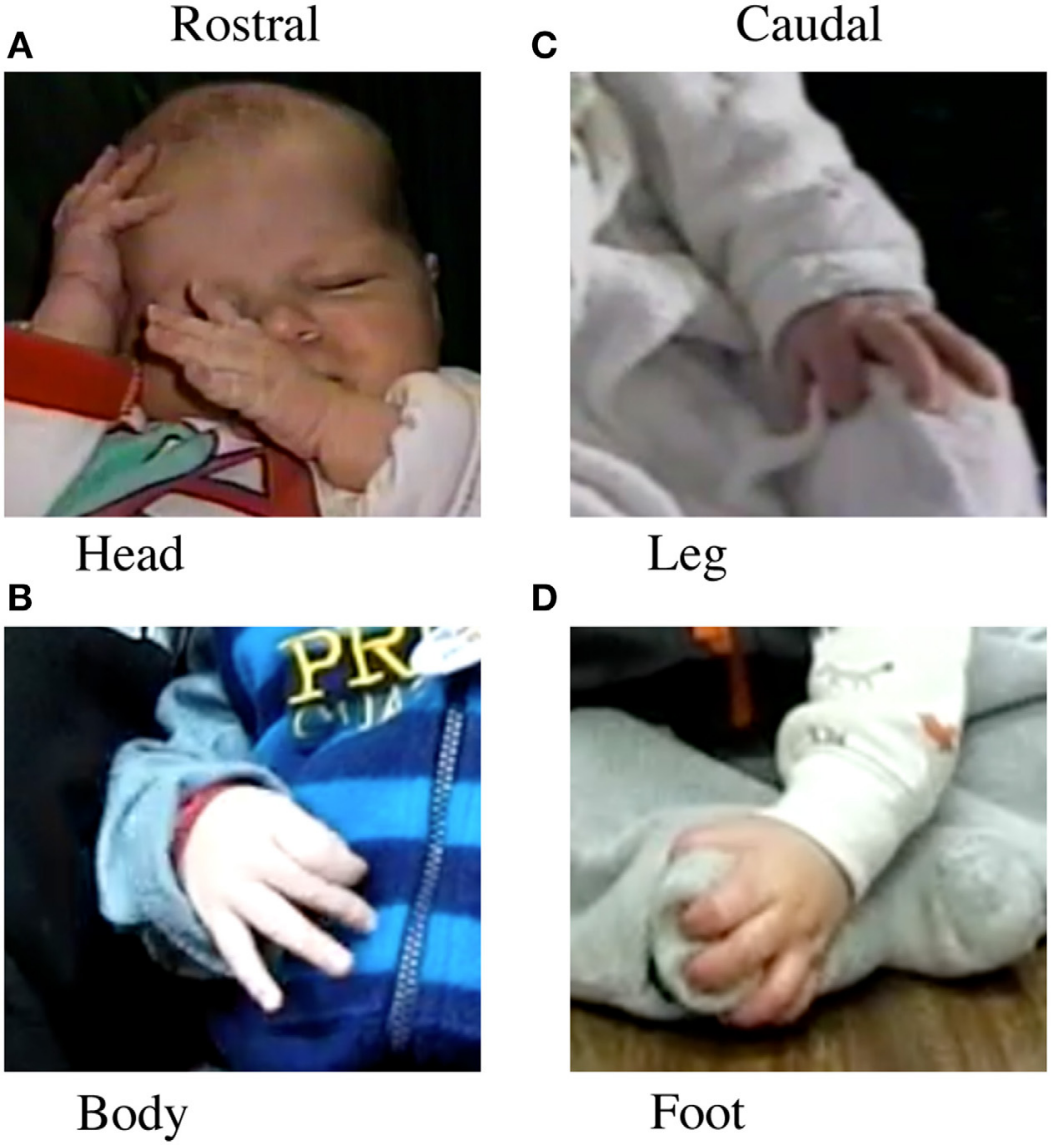
**Location of body contact. Left**. Rostral contact on **(A)** the head, **(B)** the trunk. **Right:** Caudal contact on **(C)** leg and **(D)** foot.

**Figure 2:**
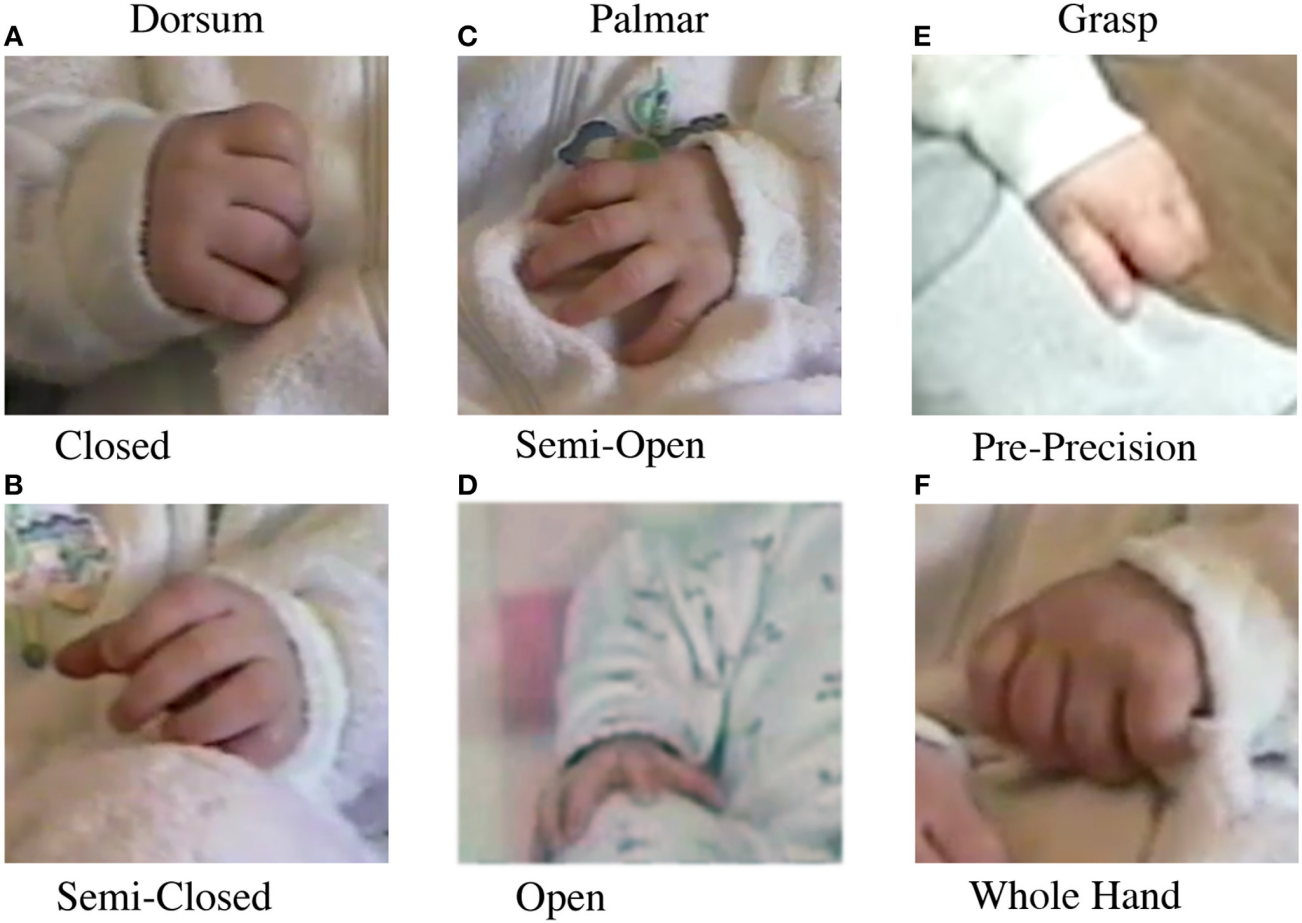
**Hand posture during contact with the body**. **Left:** Dorsum contacts, **(A)** contact with the back of the digits with the hand closed, **(B)** contact with the back of the digits with the hand semiclosed. **Middle:** Palmar contact, **(C)** contact with the digit pads and partially open hand, **(D)** contact with the fully open hand. **Right:** Grasp contacts, **(E)** pre-precision grasp between the thumb and side of the index finger, **(F)** whole hand grasp.

1. *Rostral vs. Caudal Body Contacts*. Rostral contacts (Figures 1A,B) were any self-contacts by a hand to the head, trunk, arm, or other hand. Caudal contacts (Figures 1C,D) were any self-contacts by a hand to the hips, upper leg, lower leg, or feet.
2. *Dorsum vs. Palmar.* Dorsum contacts (Figures 2A,B) were any self-contact with the dorsal aspect of the hand, including the back of the digits or the sides of the hand. Hand shapes could include a fist shape, a semi-closed hand with the thumb often tucked under or over the fingers, or an open hand. Palmar contacts (Figures 2C,D) were any self-contact with the Palmar aspect of the hand, including the fingertips, the palm, or the ventral sides of the hand. Hand shapes could include a partially open hand in which only the Palmar digit tips were in contact, or a more open hand in which the digits, palm, or digits and palm were in contact.
3. *Grasp contacts.* Grasp contacts (Figures 2E,F) were defined as the closing of one or more of the digits around the infant's body or clothing (Wallace and Whishaw, 2003). These Grasps included pre-precision grasps, in which only one or a few digits were involved in grasping, and whole hand Grasps, in which all digits were involved. A note was also made with respect to whether a grasped target was manipulated after grasping.

For each sampling period for each infant, the first 40 instances of self-touching behavior were documented, irrespective of which hand was used. The duration of the positioning of each hand movement was not noted, but most contacts were discrete in that the contact was broken shortly after it was made. One investigator (BLT) scored all of the behavior while two other investigators (JMK, LAL) scored samples of behavior in order to establish rater reliability. Inter-rater reliability for whether the hand contacted the body, whether contact was Rostral or Caudal, and whether contact was Dorsum, Palmar, or Grasp exceeded 95% agreement between the raters.

### Statistical analysis

The frequency of body contacts and hand posture contacts, as a function of infant age, were subject to statistical analyses using the computer program SPSS (v. 21.0.0.0). To accommodate uneven data points across infants, results were evaluated using repeated-measures mixed linear models (MLM; Verbeke, 2009; Heck et al., 2014). Age (0, 2, 4, 6, 8, 10, 12, 14, 16, 18, and 20 weeks) served as the within-subjects factor. A *p*-value of 0.05 was considered significant.

## Results

**Figure 3:**
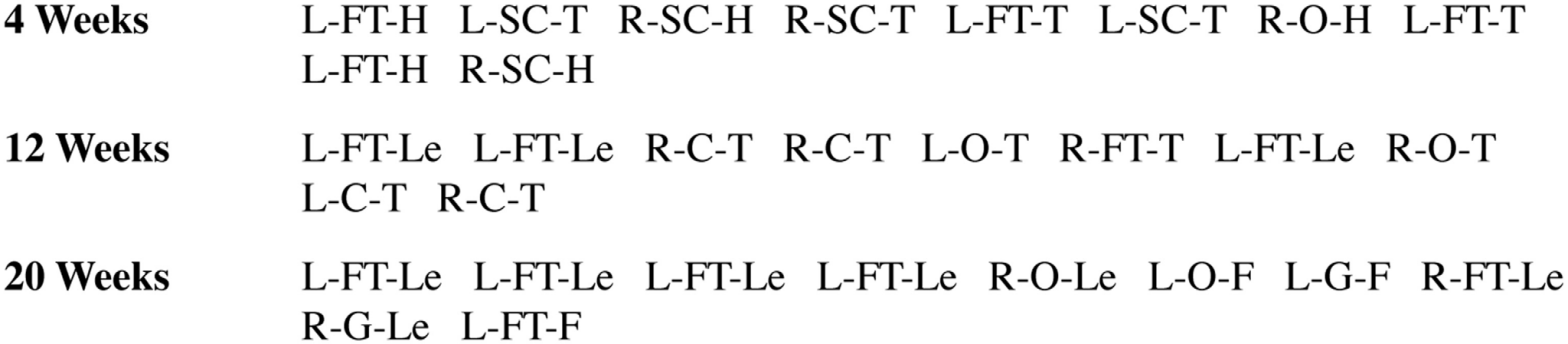
**Sample coding of the ethogram illustrating some of the hand shapes/body location of the first 10 contacts made by one infant at three different ages (1, 3, and 6 months)**. (1) Hand: R, right; L, left; (2) Hand Shape: C, closed; SC, semi-closed; FT, fingertips; O, open; (3) Location: H, head; T, torso; Le, legs; F, feet.

From birth through 6 months of age, infants displayed many spontaneous contacts of the hands with the body. The ethogram in Figure 3 illustrates a sample of the hand shapes/body location for the first 10 contacts made by infants at three different ages. Note that these samples were collected in an average of 21 s of observation time at each age (10–39 s). There was no evidence for differences in the location or hand posture of contacts according to hand or sex. Thus, sex and hand were compiled in the results. Infants ages 20, 22, and 24 weeks were also combined for this analysis, as behavioral results were asymptotic for these ages. Overall, the results show that there is a developmental transition from Rostral to Caudal contacts, a developmental transition from Dorsum to Palmar contacts, and a developmental point at approximately 16 weeks of age at which infants show an increased proportion of Grasp contacts.

### Rostral vs. caudal body contacts

**Figure 4:**
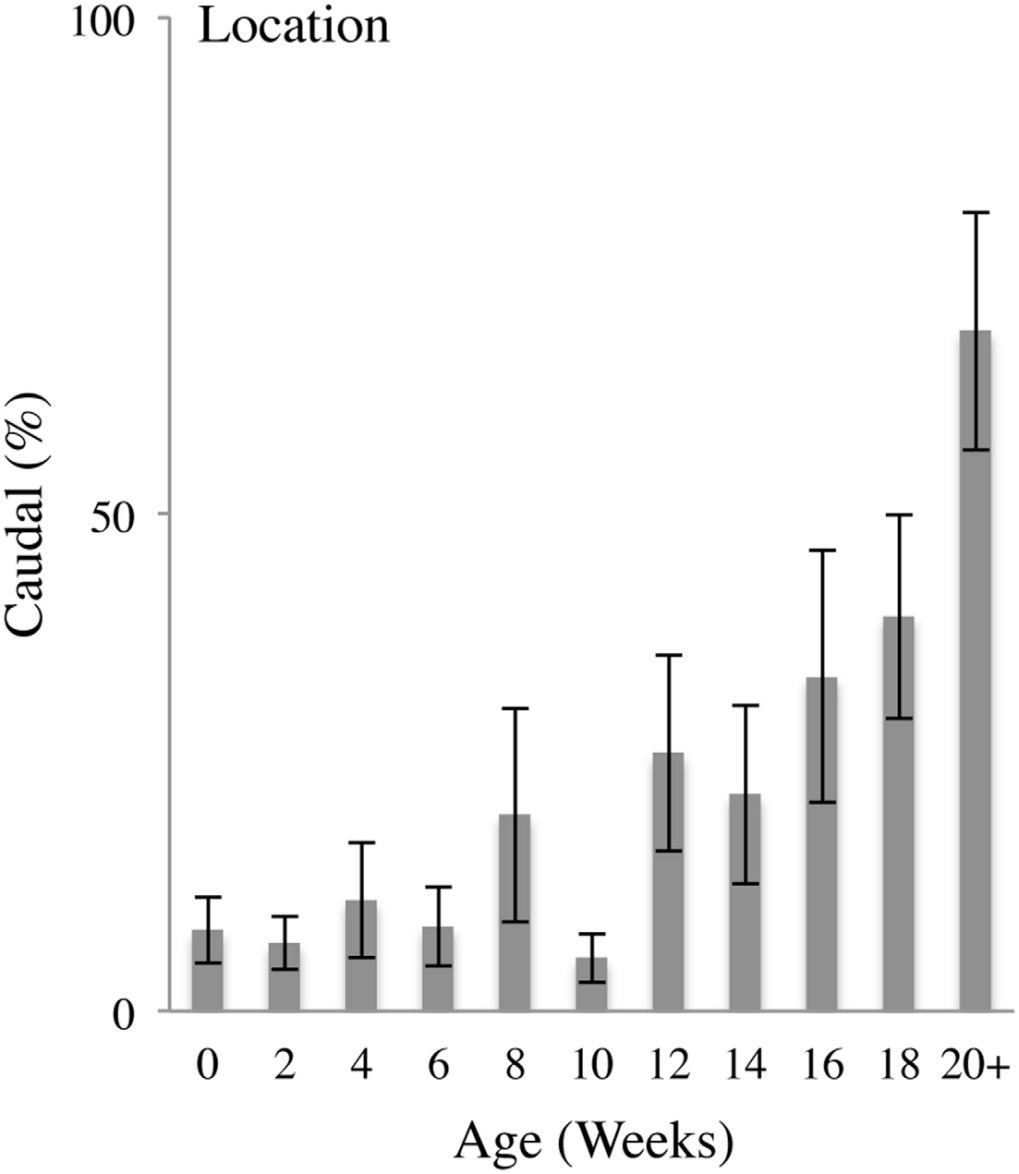
**Caudal body contacts**. Percent (mean and standard error) of contacts with the hand on the Caudal portions of the body (legs and feet) relative to all contacts as a function of age.

Figure 4 illustrates the percent of hand-to-body contacts to the Caudal portions of the body (legs and feet) as a function of age (Video 1). In the earliest weeks, the infants mainly made contacts to the Rostral region of the body, including the head, torso, arms and hands. Rostral hand-to-body contacts were restricted to the areas of the body within immediate proximity of the hand. And so, for an arm that was largely flexed at the elbow, contact was made with the head or torso. At approximately 12 weeks of age onwards, increased numbers of contacts were made with Caudal regions of the body (including the hips, legs and eventually the feet). Caudal hand-to-body contacts began with contacts to the hips and upper thighs, and expanded toward the knees and feet at approximately 20 weeks of age. Hand-to-body contacts with the knees and feet frequently involved bending of the knees and bringing the feet up toward the torso, especially when the infant was lying on his or her back.

In sum, Caudal contacts began to occur with increasing frequency at approximately 14 weeks of age, progressing from contacts with the head and trunk to contacts with the hips, legs, and feet. Thus, as a proportion of all body contacts, Caudal contacts increased as a function of age as indicated by a repeated measures MLM for Caudal contacts that gave a significant effect of Age [*F*_(10, 13.037)_ = 5.633, *p* < 0.01]. *Post-hoc* comparisons revealed that, compared to 0 weeks of age, the percentage of Caudal contacts was significantly increased at 14 (*p* < 0.05), 16 (*p* < 0.05), 18 (*p* < 0.01), and 20+ (*p* < 0.001) weeks of age.

### Dorsum vs. palmar contacts

**Figure 5:**
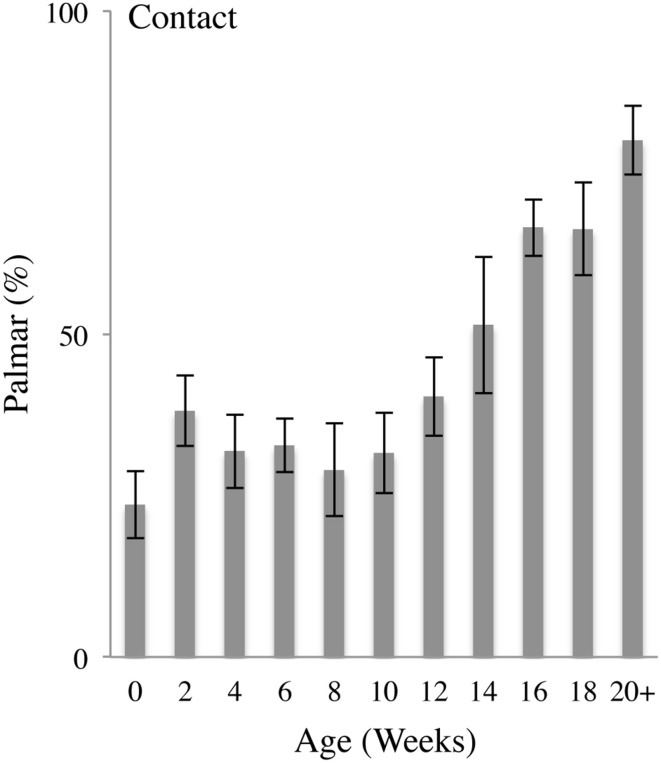
**Palmar body contacts**. Percent (mean and standard error) of Palmar contacts (contact with the digit pads or palm) relative to all contacts as a function of age.

Figure 5 illustrates the percent of Palmar contacts as a function of age. In the earlier weeks, the infants mainly contacted the body using the Dorsum of the hand, with a high frequency of self-contacts made with a fist, progressing to Dorsum contacts with a semi-closed hand, including contacts with the back of the fingers and the side of the hand. Duration of hand-to-body contact length was brief, marked mainly by contact and release (Video 2). At 8–12 weeks, hand-to-body contacts become increasingly exploratory with increased contact duration, digit manipulation, and movement. By 12 weeks Hand-to-body contacts were increasingly made with the Palmar aspect of the hand and became more complex, often involving rotation of the hand at contact, dragging the palm or fingertips along the surface of the body, and dynamic and complex hand shaping sequences.

In sum, Palmar contacts began to occur with increasing frequency at approximately 12 weeks of age, progressing from contacts with the pads of the fingertips, to dynamic contacts with the open palm. As a proportion of all body contacts, Palmar contacts increased as a function of age as indicated by a repeated MLM for Palmar contacts that gave a significant effect of Age [*F*_(10, 20.125)_ = 7.092, *p* < 0.001]. *Post-hoc* comparisons revealed that, compared to 0 weeks of age, the percentage of Palmar contacts was significantly increased at 12 (*p* < 0.05), 14 (*p* < 0.05), 16 (*p* < 0.001), 18 (*p* < 0.001), and 20+ (*p* < 0.001) weeks of age.

### Grasp contacts

**Figure 6:**
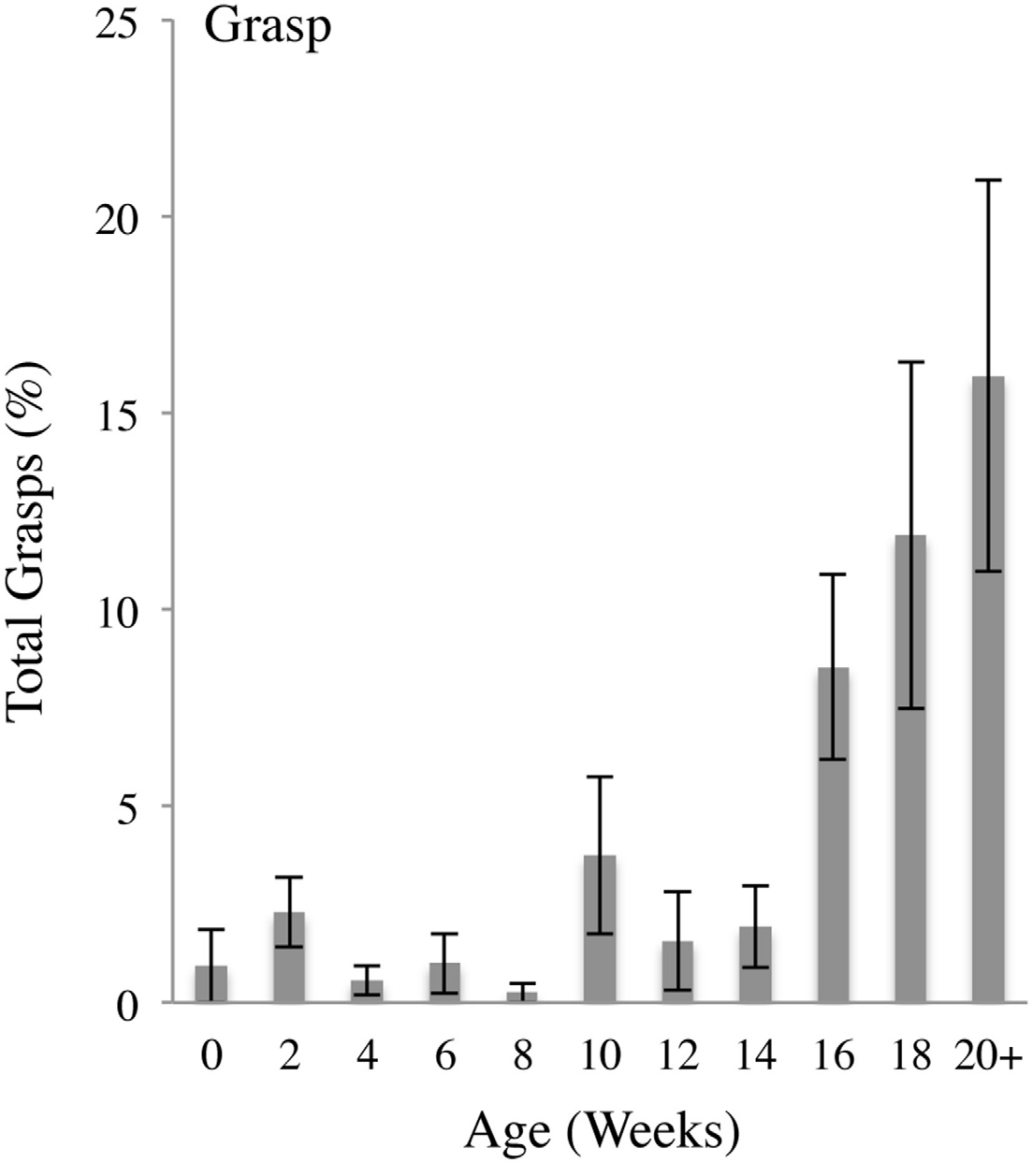
**Grasp body contacts**. Percent (mean and standard error) of Grasp contacts (pre-precision or whole hand) relative to all contacts as a functions of age.

Figure 6 illustrates that the incidence of Grasps as a percentage of all hand contacts was low in infants aged 0–14 weeks and then increased at 16–20 weeks. The self-directed preGrasps that occurred within the first week of infancy continued to occur at a relatively low frequency across the 24 weeks of study whereas whole hand Grasps became prominent at 16 weeks of age. As a proportion of all body contacts, Grasp contacts increased as a function of age as indicated by a repeated MLM for Grasp contacts that gave a significant effect of Age [*F*_(10,10.547)_ = 3.935, *p* < 0.05]. *Post-hoc* comparisons revealed that, compared to 0 weeks of age, the percentage of Grasp contacts was significantly increased at 16 (*p* < 0.05), 18 (*p* < 0.05), and 20+ (*p* < 0.05) weeks of age.

### Developmental patterns

**Figure 7:**
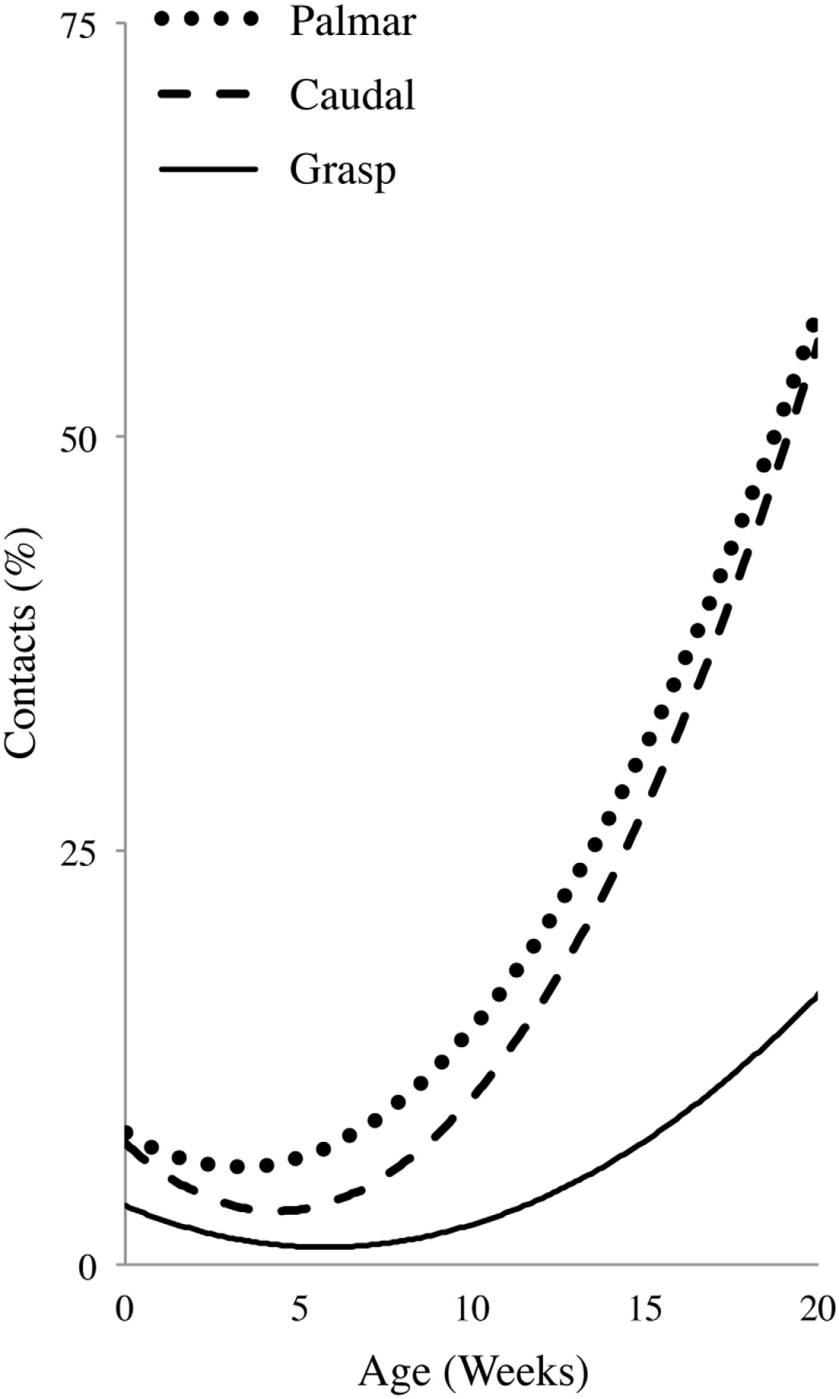
**First order polynomial regression illustrating the developmental profile of hand-to-body Palmar contacts, contacts to the Caudal region of the body, and Grasps**. Note the differences between Palmar/Caudal contacts and Grasps.

Normalized regression curves for Caudal, Palmar, and Grasp contacts are shown in Figure 7. Spearman's correlations gave a significant Caudal vs. Palmar Rho = 0.806 (*p* = 0.005), a significant Palmar vs. Grasp Rho = 0.770 (*p* = 0.009), but no Caudal vs. Grasp Rho = 0.503 (*p* = 0.138). The regression curves suggest that increases in Caudal and Palmar contacts are of a comparable magnitude and follow a similar developmental time course. By contrast, the regression curve for Grasps is reduced and shifted to the right, indicating that the incidence of Grasps did not become prominent until somewhat later. These relations are also reflected by follow-up tests described in the Dorsum-Palmer, Rostral-Caudal, and Grasp sections above. The significant relationship between Grasp and Palmar is likely due to the fact that a Grasp is dependent upon a Palmar contact.

## Discussion

There are two novel contributions of this study. First, it was found that otherwise resting infants in the first 6 months of life made many, almost continuous, forelimb movements that resulted in hand contacts with the body. These contacts eventually included grasping and manipulating the body and clothes in all regions of the body. Thus, self-touching behavior in infants is revealed to be a behavior in which infants can practice reaching, and perhaps additionally acquire body awareness in relation to a hand-related schema. Second, the analysis of self-touching movements suggests that advancing the hand to different body targets and contacting the body with the digit tips and palm represent an early developmental phase of the Reach whereas grasping the body and clothes and performing manipulatory movements represent an early phase of the Grasp. Because Reach activities developmentally preceded Grasp activities, the results suggest some independence of the two movements. Taken together with previous work showing that infants do not need to view their own hand in order to transport it to a target (Clifton et al., 1993; Corbetta, 2010), the timing and the sophistication of hand contacts with the body observed in the present study suggest that reaching undergoes substantial preparedness under the auspices of proprioception and touch prior to and in concert with the emergence of visually guided reaching.

It is important to note that the present study was primarily directed toward describing self-touching hand movements and secondarily at assessing the idea that during development there is some independence in the display of reaching and grasping movements as has been suggested in studies largely directed toward visually guided reaching (Von Hofsten and Lindhagen, 1979; Trevarathen, 1982; von Hofsten, 1984; Savelsbergh and van der Kamp, 1994; Wimmers et al., 1998a,b). Thus, although it is obvious that the spontaneous activity that we have observed is likely the result of interactions between nervous system development, morphological development of the body, the posture of the infants during testing, and the life history of the experimental subjects (Savelsbergh and van der Kamp, 1994; Thelen and Spencer, 1998; Heathcock et al., 2004; Lobo et al., 2014; Soska and Adolph, 2014), there was no intent in the present study to distinguish between these contributing factors. Rather, it was our view that any differences in the developmental profile of reaching and grasping might contribute to a growing body of evidence that the Reach and the Grasp are mediated by different sensorimotor channels (for a review of other infant work directed toward this question see Karl and Whishaw, 2014). As noted by Hebb (1949) “The problem of understanding behavior is the problem of understanding the total nervous system and *visa versa (xiv)*.”

Specifically, three aspects of hand-to-body contact were documented in relation to infant age: an increasing incidence of caudal body relative to rostral body contacts, an increasing incidence of palmar relative to dorsum hand contacts, and an increasing incidence of contacts that resulted in Grasps of the body and clothes. An increase in the incidence of palmar and caudal contacts occurred at a somewhat earlier age than did the increase in the incidence of Grasps. Because the Reach in adults is associated with forelimb movement and a more open hand to make palmar contact with a target, we suggest that the forelimb movement and palmar contact in infants is a manifestation of an infant Reach. Because the Grasp in adults includes digit flexion and closing to purchase and manipulate an object, we suggest that self-grasping in infants is an early manifestation of an infant Grasp. Thus, we suggest that the developmental pattern of these Reach and Grasp movements in infants supports the Dual Visuomotor Channel Theory, which proposes that the reaching act is enabled by separate Reach and Grasp neural systems. Of course, morphological development including increases in the length of the arms, the size of the hands, and body strength in all likelihood are also necessary for some part of the maturation of the movements. Nevertheless, the hand to body self-touching movements seen in the infants likely continue throughout life and likely continue to serve some of the same purposes in adults that they serve in infancy.

The design of the present experiment is similar to that of a number of our previous studies in that it is ethological, focuses on infant spontaneity, and searches for structural organization within this activity. It also featured a number of procedures to ensure accurate measurement of spontaneous hand-to-body contacts in infants (Wallace and Whishaw, 2003; Sacrey and Whishaw, 2010; Foroud and Whishaw, 2012). First, toys and other distractions were removed to ensure that self-directed movements were unbiased by extraneous influences. Second, to control for individual differences in the frequency of hand-to-body contacts, 40 consecutive contacts within each 10-min recording period were used for analysis. Third, high inter-rater reliability scores among 3 independent raters on the main behaviors that were measured confirmed the validity of the scoring method. These procedures ensured that the infants were similarly relaxed and alert and otherwise not disturbed and so were likely to engage in a common class of relatively spontaneous activities across the study period.

In many respects, this work differs from the more formal studies of visually guided reaching in which both the task and the outcome are constrained. For example, in the Wimmers studies (Wimmers et al., 1998a,b), described in the introduction, infants are encouraged to purchase a proffered object, resulting in seemingly age-related dichotomous behavior, reaching without grasping followed by reaching with grasping. Spontaneous self-directed movements of the hand described here also reflect a developmental profile in which the Reach matures before the Grasp, but one behavior does not completely replace the other. The spontaneous manual interaction with objects when documented in a ethological context also suggests that reaching without grasping and reaching with grasping co-occur (Lobo et al., 2014). Although the present study was not directed at examining how reaching and grasping occur, work with older infants suggests that there is a very prolonged developmental period, likely lasting beyond 2 years of age, in which the Reach and Grasp are not yet fully mature and not yet fully integrated (Karl and Whishaw, 2014). Further work using high speed filming of infant self-grasping could be used to examine the detailed architecture of the Reach and the Grasp in self-grasping because it might be expected that online somatosensory guidance of reaching matures before the online visual guidance of reaching (Karl et al., 2012b).

A number of caveats in relation to the present methods must be noted. First, infants were filmed in a variety of settings including the home and laboratory, the time of day during which filming occurred was variable, and the postures of the infants did vary somewhat depending upon their age, and all infants could not be filmed at every age. It might be considered, however, that such variation strengthens the ethological relevance of the sampling method. Second, infants were usually clothed and so it was not possible to confirm that similar hand-to-body behavior would be demonstrated in the absence of clothing. For example, the presence of clothing might serve to encourage grasping behavior. It was noted, however, that there were no obvious differences in the behavior of infants for whom clothes were tight fitting versus loose fitting. Third, the sampling periods were limited to resting behavior and did not include other activities, including breast or bottle feeding or interpersonal play, which could provide additional information concerning the development of hand contacts to the self and proximal objects. In addition, the infants' spontaneous activity included many other activities such as movements of the head, trunk, and legs and these activities were not documented. Nevertheless, the high number of hand-to-body contacts that occurred in each infant and the systematic changes in the location and way that the hand contacted the body across the developmental period examined suggests that this data sample is sufficiently robust to provide insights into an activity that must occur in infants many hundreds of times each day.

There are a number of features of the present results that we feel justify concluding that they reveal a novel insight into the developmental progression of reaching behavior and its relation to the distinctive Reach and Grasp movements of adults as characterized by the Dual Visuomotor Channel Theory. First, studies that have manipulated the visual contribution to reaching show that without vision the Reach consists of a movement of extending the arm and hand with open digits in order to make palmar contact with a target (Karl et al., 2012a; Karl and Whishaw, 2013). We suggest that in infants, the development of hand-to-body contacts from rostral to caudal body locations associated with the increasing frequency of opening the hand to make palmar contacts might be a developmental precursor of the adult manifestation of the Reach. That is, in the initial weeks of the samples, arm movements were largely movements around the shoulders with the digits in a mainly closed configuration (Sacrey and Whishaw, 2010) that resulted in incidental hand to body contact. Eventually, the arm movements included movements of the trunk and all of the forelimb joints, including extension of the digits. In doing so, they included palmar contact that began to have an exploratory character and that increasingly included the caudal regions of the body. The movements also became coordinated with other body movements as exemplified by reaches that contacted the feet and toes that were themselves in motion. It is also noteworthy in this respect that regression profiles of touches on caudal body locations and the use of palmar contacts were very similar. Thus, in their eventual configuration, infant reaches to touch the body resembled the Reach made by unsighted adults in that the arm carries an open hand to make a palmar contact with a target.

It is interesting that Pellijeff et al. (2006) show that reaches made by adults to their own hand, located near their own torso, are associated with fMRI activation in the cortical area of the anterior precuneus and medial intraparietal sulcus in the superior parietal lobe. This is the same region that is activated for both proprioceptively and visually guided reaching toward external objects (Filimon et al., 2009). Therefore, we suggest that infant reaches toward the torso and body are analogous to adult reaching to distal targets, adding support to our suggestion that caudal directed reaches and touches serve as a developmental precursor/addition to reaching to visual targets.

We were, of course, unable to determine the extent to which reaches to various body parts were vacuous versus goal directed but we propose that the scope and frequency of the movements provides ample room for arm movements to mature both in their configuration (von Hofsten, 1984) and intent (Lew and Butterworth, 1997). We note that after palmar contacts begin to occur they also begin to take on an exploratory character in frequently caressing the part of the body that is contacted. As such, the practice/development of these movements made to body targets might well be preparatory/facilitatory for reaches that will subsequently be directed to targets during visually guided reaching (White et al., 1964; McDonnell, 1975; von Hofsten and Fazel-Zandy, 1984; von Hofsten and Ronnqvist, 1988; Lobo et al., 2004; Lobo and Galloway, 2013). In the present study, we observed few movements directed toward the mouth, and accordingly did not separately document them, but other research has found that these movements only become frequent after about 4 months of age, an observation consistent with the present results that it is at about this age that hand movements are becoming goal directed (Lew and Butterworth, 1997; Sacrey et al., 2012).

According to the Dual Visuomotor Channel Theory, the Grasp preshapes the digits relative to target size and adjusts the digits for appropriate target purchase (Arbib, 1981). In the absence of vision, shaping and grasping are instructed by haptic information provided by touch (Karl et al., 2012a; Karl and Whishaw, 2013). In the infants examined in the present study, the first grasps featured hooking one or another digit into the clothing, they then involved clasping with the thumb or other digits, and by the end of the observational period they featured whole hand grasps that included manipulation. We suggest that this pattern features a progression in “maturation and learning to grasp.” Our observations and interpretation are consistent with an extensive literature on infant and fetal hand use (Twitchell, 1965; Hepper, 1990; Hepper et al., 1991; Sparling and Wilhelm, 1993; Sparling et al., 1999). Nevertheless, prior to the various grasping acts, there was no obvious shaping of the digits prior to target contact nor was obvious hand shaping present between successive contacts. The absence of digit preshaping is not surprising because evidence from studies on the development of visually guided reaching suggests that hand preshaping continues to mature beyond 2 years of age (McCarty et al., 2001; Karl and Whishaw, 2014).

Evidence that grasping movements have a partially different developmental onset than reaching movements was supported by our finding that the developmental profile of grasping frequency was statistically unrelated to the Rostrocaudal profile of body contact and was only somewhat weakly related to the Dorsopalmar profile of hand contact, which were themselves tightly coupled. That is, the onset of frequent self-grasping occurred at a somewhat later age than the onset of frequent caudal body contacts and palmar contacts. We suggest that this difference provides further support for the idea that the Reach and the Grasp have different developmental onset. That is, our results suggest that the Reach, consisting of an ability to move the hand to a body target with the digits open to make a Palmar contact with the target, is achieved before the hand begins to engage in substantial object purchase, which characterizes the Grasp. Of course, the movements are not completely unrelated because a Reach with Palmar contact necessarily precedes a Grasp. Nevertheless, it is interesting that an examination of the early development of visually guided reaching similarly suggests that Reach maturation precedes Grasp maturation (Karl and Whishaw, 2014; see also Von Hofsten and Lindhagen, 1979; Trevarathen, 1982; von Hofsten, 1984; von Hofsten and Fazel-Zandy, 1984; Ruff, 1989; Savelsbergh and van der Kamp, 1994; Wimmers et al., 1998a,b; Corbetta and Snapp-Childs, 2009).

In previous work, we have suggested that the Reach and the Grasp have different evolutionary origins, the Reach derived from stepping and the Grasp derived from food handling movements (Karl and Whishaw, 2013). In light of this suggestion, the present findings might seem surprising because the development of self-touching Reach and Grasp movements occur both before the onset of walking (crawling) and the onset of hand use for self-feeding. In humans, however, self-feeding and walking are developmentally delayed. It is possible that the many leg movements associated with self-directed reaches to the caudal body are a developmental precursor for walking and may facilitate the development or refinement of neural circuitry in the superior parietal lobe that is common to both stepping and reaching (Bakola et al., 2010, 2013; Karl and Whishaw, 2013). Although leg movements were not analyzed in the present study, the relationship between arm movement and leg movement could be addressed by examining their relationship in human infants as well as their early development in other animal species, especially other primate species (e.g., Wallace et al., 2006). Similarly, hand movements in infants are often associated with mouth movements (Iverson and Thelen, 1999). It is possible that the species-typical developmental profile of humans results in suppression and reordering of the development of many movements (Schott and Rossor, 2003).

Speculatively, the present results could be related to the Dual Visuomotor Channel Theory in other ways, including the establishment of body spatial schema and hand action schema related to objects (Granmo et al., 2008; Yamada et al., 2013). In this respect it is relevant that the Reach is importantly directed to the extrinsic (e.g., location) properties of targets using egocentric coordinates provided by proprioception. Early prereach activity associated with self-touching could contribute to the development of egocentric coordinate systems. It is also relevant that the Grasp is importantly guided by the intrinsic properties (size, shape, etc.) of a target. Infant self-grasping acts could contribute to the development of a hand schema that provides an appreciation for the intrinsic properties of objects. Because both the body and hands are undergoing continuous morphological change (Newell et al., 1989, 1993), the high incidence of self-touching and grasping could contribute to updating hand and body schema.

In summary, developmental research presupposes that developing actions are the foundation for more complex adult behavior (Lobo and Galloway, 2008) and that development frequently has a proximodistal progression (Berthier et al., 1999). Although numerous hand-to-body contact behaviors and hand manipulative capabilities have been observed in development, including in fetal development (Hepper, 1990; Hepper et al., 1991; Sparling and Wilhelm, 1993; Sparling et al., 1999), the present results are consistent with these general sequences and also offer two new insights into the development of reaching. First, we suggest that hand-to-body contact is a formative stage in the development of the adult Reach. It is likely that the maturation of self-contact movements into self-grasping movements is an important preparatory stage for the development of the adult Grasp. Second, we suggest that the early development of arm movement and hand touching compared to the later development of the pattern of self- grasping and manipulation provide evidence that the Reach and the Grasp have at least partially separate developmental profiles. Finally, we suggest that the development of the Reach and the Grasp and their integration is importantly related to practice provided by the high incidence and changing patterns of hand self-contact behavior.

### Conflict of interest statement

The authors declare that the research was conducted in the absence of any commercial or financial relationships that could be construed as a potential conflict of interest.
